# EPELI: a novel virtual reality task for the assessment of goal-directed behavior in real-life contexts

**DOI:** 10.1007/s00426-022-01770-z

**Published:** 2022-11-23

**Authors:** Erik Seesjärvi, Jasmin Puhakka, Eeva T. Aronen, Alexandra Hering, Sascha Zuber, Liya Merzon, Matthias Kliegel, Matti Laine, Juha Salmi

**Affiliations:** 1 Department of Psychology and Logopedics, University of Helsinki, P.O. Box 4, 00014 Helsinki, Finland; 2 Child Neurology, University of Helsinki and Helsinki University Hospital, Helsinki, Finland; 3 Child Psychiatry, University of Helsinki and Helsinki University Hospital, Helsinki, Finland; 4 Laboratory of Developmental Psychopathology, New Children’s Hospital, Pediatric Research Center, Helsinki, Finland; 5 Department of Developmental Psychology, Tilburg University, Tilburg, The Netherlands; 6 Swiss National Centre of Competence in Research LIVES, Geneva, Switzerland; 7 Institute on Aging and Lifelong Health, University of Victoria, Victoria, Canada; 8 Department of Psychology, University of Geneva, Geneva, Switzerland; 9 Department of Psychology, Åbo Akademi University, Turku, Finland; 10 Department of Neuroscience and Biomedical Engineering, Aalto University, Espoo, Finland; 11 MAGICS, Aalto University, Espoo, Finland

## Abstract

A recently developed virtual reality task, EPELI (Executive Performance in Everyday LIving), quantifies goal-directed behavior in naturalistic conditions. Participants navigate a virtual apartment, performing household chores given by a virtual character. EPELI aims to tap attention, executive function, and prospective memory. To ensure its applicability to further research and clinical work and to study its relationship to relevant background factors, we examined several key properties of EPELI in 77 typically developing 9–13-year-old children. These included EPELI’s internal consistency, age and gender differences, sensitivity to gaming experience, head-mounted display (HMD) type, and verbal recall ability, as well as its relationships with parent-rated everyday executive problems. Of the eight EPELI measures, the following six showed acceptable internal consistency: task and navigation efficacy, number of correctly performed tasks and overall actions, time monitoring, and controller movement. Some measures were associated with age, gender, or verbal encoding ability. Moreover, EPELI performance was associated with parent-rated everyday executive problems. There were no significant associations of gaming background, task familiarity, or HMD type with the EPELI measures. These results attest to the reliability and ecological validity of this new virtual reality tool for the assessment of attention, executive functions, and prospective memory in children.

**Supplementary Information:**

The online version contains supplementary material available at 10.1007/s00426-022-01770-z.

## Introduction

In cognitive psychology and neuropsychology, there is a long history of using experimental paradigms that contain a relatively narrow set of stimuli and allow for only a limited range of behavioral responses from the experimentee (Hatfield, 2002). The use of such paradigms and measures has unarguably yielded a wealth of information on human cognition in both healthy participants and clinical groups. However, some critics have questioned whether this approach alone is sufficient when the subject of the study is something as complex as human goal-directed behavior taking place in complex everyday situations (e.g., Bronfenbrenner, 1977; Brunswik, 1943; Gibson, 1970; Kingstone et al., 2008; Neisser, 1976). In particular, there has been a call to develop neuropsychological tests with increased ecological validity, that is tasks which are more closely related to everyday tasks (Franzen & Wilhelm, 1996).

There are two general types of ecological validity of a test measure to consider here, namely verisimilitude and veridicality. Verisimilitude refers to how accurately the test properties resemble corresponding situation in the real world, whereas veridicality refers to the extent to which results on a test reflect or predict the skills or task performances in everyday life (Franzen & Wilhelm, 1996). There are different approaches to assess and improve the verisimilitude and veridicality of a test (Chaytor & Schmitter-Edgecombe, 2003; Chaytor et al., 2006; Parsons, 2015). In some studies, cognitive functions have been measured “in the wild”, by instructing the participants to perform specific cognitively demanding tasks, for instance, in a shopping street (Garden et al., 2001; Shallice & Burgess, 1991). As these tasks take place in real-life environments, they have high verisimilitude (Spooner & Pachana, 2006). However, it is difficult to control for the complex environmental effects in real-life conditions, and the precise measurement of behavior becomes challenging too. Therefore, this type of conditions may suffer from limited reliability and participants safety might also be compromised (Logie et al., 2011). Furthermore, taking the testing to actual real-world environments can be time-consuming and impractical. Another, more convenient option is to include features to a computer task that will increase its ecological validity. For instance, in the Virtual Week task, participants are asked to perform a set of everyday tasks in a board game (Rendell & Craik, 2000). This type of tasks can be considered to share higher resemblance with real-life situations than conventional paper-and-pencil tasks or experimental cognitive psychology tasks with restricted stimuli, but it is debated whether such tasks measure the same cognitive processes that are required in real-life situations (Parsons, 2015). In computerized tasks, the interaction with the environment is typically quite artificial and time scale of task performance is unrealistic. Moreover, even though a structured task has benefits raising from clearly defined measures, it can be criticized that function-led tasks aiming at high ecological validity (e.g., the Virtual Week) may not generalize to multiple different situations. This is the aim with conventional construct-laden neuropsychological tasks, such as Wechsler’s Intelligence Scale for Children, that are designed to tap several core cognitive functions reflecting general abilities (Wechsler & Kodama, 1949). All in all, there are multiple difficult choices that the experimenter needs to make to select an appropriate task, which will influence how well the outcome measure can be defined and how accurately does the respective measure reflect something being actually done in everyday life (Iverson et al., 2008).

Virtual reality (VR) provides means to improve the ecological validity of a psychological test without compromising the experimental control considerably. VR refers to using digitally generated, artificial environments to recreate real-world activities to participants. When presented with 2D computer screens, VR can be referred to as non-immersive, whereas immersive VR can be achieved with head-mounted displays (HMDs), that offer extended field of view and stereoscopic vision (Parsons, 2015). Immersive VR has been applied in neuropsychological research targeting, for example, attention, executive functions, and memory (see review by Kim et al., 2021), and it is gradually becoming more popular as research and clinical tool in the field of neuropsychology (Krohn et al., 2020). In children, HMD-VR has been successfully used to implement conventional experimental tasks, such as a variant of the widely used Continuous Performance Test where the task is performed in a virtual classroom environment (see, e.g., Parsons & Rizzo, 2019). One major benefit of VR is that it also allows the creation of complex, realistic everyday scenarios while simultaneously providing precise control over test parameters and measurements (Parsons et al., 2017). This makes VR an attractive way to pursuit the development of more ecologically valid tests (Parsons, 2015). Indeed, in adult populations, several VR tasks that simulate common everyday activities (e.g., grocery shopping or cooking) with immersive HMDs have already been successfully employed (Barnett et al., 2021; Chicchi Giglioli et al., 2021; Ouellet et al., 2018). A more comprehensive endeavor was taken up by Kourtesis et al. (2020), who developed *VR-EAL*, a VR-based neuropsychological battery for adults that lasts about 60 min and consists of multiple scenes with real-life elements. The participants rated VR-EAL as more similar to everyday tasks compared to equivalent ecologically valid paper-and-pencil tasks (Kourtesis et al., 2021), which attests to the potential of VR paradigms to reach high verisimilitude. Taken together, these studies point to the feasibility of immersive VR as a versatile medium for creating more realistic neuropsychological measures. However, especially for children there is a lack of naturalistic tasks, which would, for example require the participants to engage in goal-directed behavior in open-ended, likelike situations.

To study the goal-directed behavior of children in everyday context, we developed *EPELI* (*Executive Performance in Everyday LIving*; Seesjärvi et al., 2022). To our knowledge, EPELI is the first HMD-based VR task that requires children to plan and carry out multiple tasks by navigating a virtual environment and interacting with the relevant target objects there, while monitoring the time and ignoring distracting objects and events. The development of EPELI was inspired by naturalistic prospective memory studies (Rendell & Craik, 2000) and studies carried out in real-life environments, such as *Multiple Errands Test* (Shallice & Burgess, 1991). In EPELI, children perform short scenarios consisting of everyday chores in a virtual apartment. Each scenario starts with an encoding phase during which a dragon character tells the child what tasks need to be done next. In the subsequent execution phase, the goal of the child is to perform the given tasks by navigating the apartment and interacting with the relevant objects. Successful performance in EPELI requires a diverse set of attentional-executive functions (e.g., visual search, selective attention, inhibition, planning, decision making, working memory, multitasking) and prospective memory, the ability to remember and execute delayed intentions in the future (Kliegel et al., 2008). Simulating everyday situations and contexts has been suggested to overcome the key limitations of simplified laboratory tasks discussed above (e.g., Burgess et al., 2006; Chaytor et al., 2006; Dawson & Marcotte, 2017; Miller & Barr, 2017). In our first paper with EPELI that compared ADHD and typically developing children, the task showed predictive validity as the ADHD group exhibited higher percentage of irrelevant actions reflecting lower attentional-executive efficacy and more controller movements and total game actions, both possibly being indicative of hyperactivity-impulsivity (Seesjärvi et al., 2022). EPELI performance was also linked to ADHD symptoms and successfully discriminated between ADHD and typically developing children. Moreover, EPELI performance correlated strongly with parent evaluations of everyday executive problems as evaluated across all participants, a finding that provides support for its veridicality. Moreover, the participants on average gave highly positive ratings regarding the EPELI playing experience and very few participants reported simulator sickness symptoms or usability problems.

When examining VR for the development of new neuropsychological tasks, several possible confounding factors regarding both participants’ previous experience and the hardware in use should be considered. First, the gaming background could affect task performance, as experience with action video games is linked to higher performance in several cognitive domains as measured with typical computerized paradigms (Bediou et al., 2018). However, as regards to immersive VR paradigms that provide better comparison to EPELI than simplified non-immersive paradigms, Kourtesis et al. (2021) found no performance differences between gamers and non-gamers in VR-EAL, even though gamers completed the test battery more quickly than non-gamers. These findings are in line with the literature suggesting that gamers display improved perceptual processing speed (Bediou et al., 2018). Although the performance speed was not addressed in the present study, each scenario has a maximum duration of 90 s, which could lead to participants with regular gaming background having an advantage over non-gamers. Second, if the paradigm simulates real-life situations and tasks with rich and highly meaningful contents, which is the case with EPELI, differences in participants’ familiarity with the tasks and stimulus objects could affect their performance. Familiarity of the contents is considered to be a major factor especially in memory studies (e.g., Dalton, 1993; Gagné et al., 1985). Third, new VR hardware is released annually, and it should be investigated whether the technological solution could affect the results. For example, higher display resolution could make object more easily distinguishable and therefore improve performance, and using an HMD that is not only limited to tracking head rotation but tracks also head movement could provide more immersive experience. Although earlier generations of HMDs have been reported to cause simulator sickness, adverse effects or dropouts seem to be rare in studies that employ newer HMDs (see review by Kourtesis et al., 2019). Nevertheless, the possibility of sickness symptoms is still an important factor to consider in VR studies, even though it is unlikely that it would continue to hinder the development of VR-based neuropsychological tasks.

The present study examined several key properties of EPELI in a sample of 77 typically developing children of middle school age. The aims of this study include probing EPELI’s key psychometric properties, internal consistency, veridicality and verisimilitude. These properties are essential for any neuropsychological instrument to demonstrate that it is able to provide robust and generalizable results. Moreover, we wanted to study the role of age and gender in the development of real-world goal-oriented behaviors that EPELI measures. Previous studies examining the development of goal-directed behavior have mostly utilized isolated tasks, and it is still unclear whether the related findings generalize to open-ended situations provided in EPELI. Furthermore, the possible confounding effects mentioned in the previous paragraph need to be considered to ensure, that EPELI measures are truly capturing goal-oriented behavior in the same way across different participants and VR devices. Also, even though the two relevant phases in prospective memory, encoding and execution, have been widely studied both in children (Ballhausen et al., 2019; Mahy et al., 2014) and across lifespan (Zuber & Kliegel, 2020), it is still unclear how much the ability to encode the given instructions dictates the performance in the execution phase of an open-ended, naturalistic task such as EPELI. Finally, the study aims to further investigate the associations between parent-rated difficulties in executive function and EPELI in a sample consisting of only typically developing children, that were reported for children with ADHD and their controls in a previous study (Seesjärvi et al., 2022). Thus, the spesific aims were as follows:

(i) To evaluate the internal consistency of the EPELI measures that were created and used by Seesjärvi et al. (2022). Internal consistency was assessed for the full-length version with 13 scenarios as well as for smaller task sets, as developing a shorter version could be beneficial in situations with time constraints, such as in clinical use.
(ii) To examine the effects of age (from 9 to 13 years), gender, and their possible interaction on the EPELI measures. As executive functions (Best & Miller, 2010; Klenberg, 2015) and prospective memory (Ballhausen et al., 2019; Mahy et al., 2014; Zuber & Kliegel, 2020; Zuber et al., 2019) continue developing during the early school years and beyond, we expected to see better EPELI performances (higher total scores and efficacies) in older children within our age range. Even though the issue of gender differences in executive functions is controversial, there is some evidence that males may be more impulsive than females across the lifespan (for a review, see Grissom & Reynes, 2019). Moreover, Barnett et al. (2007) found that in 8–10 year-olds, girls have better scores on attention tasks and show less impulsivity than boys.
(iii) To test possible confounding effects associated with user experience and VR technology. User-related effects that were tested included participants’ gaming background and familiarity with the task contents. Moreover, to see if the type of the HMD would affect performance, we employed two different HMDs (*Oculus GO* and *Pico Neo 2 Eye*). This is important because VR technology is developing rapidly and new hardware updates are released annually.
(iv) To probe to what extent the ability to encode verbal instructions explains EPELI performance. To this end, children also performed an instruction recall task, in which they orally repeated a list of tasks similar to those that they executed in EPELI. Studying the associations between the instruction recall task and EPELI was expected to clarify the contributions of memory and executive processes, which are intertwined in prospective memory performance (Kliegel et al., 2008; see also Zuber & Kliegel, 2020).
(v) To explore the relationships between the EPELI measures and different aspects of parent-rated problems of executive function. In our first study (Seesjärvi et al., 2022), we showed that the EPELI measures correlate with parents’ overall evaluation of everyday executive problems. We expected to acquire similar results here. However, that analysis lumped together children with ADHD and their controls, leaving it open whether such associations would exist even within the normal variation that typically developing children exhibit. Moreover, it remained unclear to what extent more specific types of executive problems (i.e., those related to either behavioral regulation or metacognitive skills) are associated with EPELI performance.

## Materials and methods

### Participants

The participants were drawn from two samples (A and B). Sample A consisted of 68 typically developing children from our previous study (Seesjärvi et al., 2022) and Sample B of 32 typically developing children, who used different type of HMD than Sample A and thus enabled the comparison between two different HMDs. In both samples, the inclusion criteria were (a) native language Finnish, (b) the age of 9–12 years when recruited, and (c) no history of psychiatric or neurologic diagnoses or special support at school. They were recruited from schools in Kirkkonummi and Espoo, Finland, by sending advertisement letters to parents via schools’ electronic message boards and giving brief educational lectures at schools and online during which the study was mentioned. From Sample A, 11 children had to be discarded due to wrong settings and six due to technical failures. From Sample B, three children had to be discarded due to technical issues. There were no differences between the samples regarding age, gender, familial income, or average parental education (see Supplementary Appendix A). After outlier analysis (see below), the final sample consisted of 77 children (31 girls, age range 9.0–13.0 years; mean age 10.8 years; Table 1). All participants received two movie tickets each as compensation for their participation.

**Table 1:** Background characteristics of the final sample (*N* = 77)

Variable	Mean	SD
Age	10 years 9 months	1 year 1 month
Handedness (left/right)	6/71	
Gender (boy/girl)	46/31	
Parental income^a^	4.3	0.83
Parental education^b^	2.8	0.41
WISC-IV similarities^c^	11.3	2.70
WISC-IV matrix reasoning^c^	10.2	3.38
BRIEF GEC (0–144)	101.1	15.77
BRIEF BRI (0–56)	35.2	5.48
BRIEF MI (0–44)	65.4	11.64
Gaming background (regular/not)	67/10	
Familiarity of the tasks (1–7)	4.9	1.05
Simulator sickness (0–14)	0.1	0.13

^a^Before tax per adult; 1 = less than 1500 €/m, 2 = 1500–2200 €/m, 3 = 2200–3000 €/m, 4 = 3000–4000 €/m, 5 = over 4000 €/m

^b^1 = Comprehensive school, 2 = high school/vocational school, 3 = university degree or equivalent

^c^Standard score

### EPELI task

EPELI (https://aalto.cloud.panopto.eu/Panopto/Pages/Viewer.aspx?id=3eb4836f-1238-4f27-853a-ad3700745b31; for the original description, see Seesjärvi et al., 2022) was designed with equal contribution by ML, JS, and ES, and implemented by the Peili Vision Company (http://www.peilivision.fi/). Participants in Sample A used Oculus Go (2560 × 1440 resolution, 60/72 Hz refresh rate, and 101-degree field of view) and Sample B Pico Neo 2 Eye (3840 × 2160 resolution, 75 Hz refresh rate, and 101-degree field of view) HMD. The experimenter launched EPELI and followed the gameplay using a Samsung Galaxy Tab S3 tablet. The participant interacted with the objects by pointing at them with a hand controller and simultaneously pushing a button, with a thumb on Oculus Go and with an index finger on Pico Neo 2 Eye. They could interact with the movable objects by picking them up by pushing the button and releasing the object by pointing at a desired location and pushing the button again. An object that was taken to hand could be rotated by rotating the hand controller. The participants could check the time by raising the hand controller, which mimics checking the time from a wristwatch in real life. The drums in the game environment could be played by swinging the hand controller at them. Navigating was performed by pointing at a waypoint circle on the floor and pressing the button, which resulted in teleporting to that location. Motion tracking sensors in the HMD and the controller recorded the participant’s movements. There is a difference between the two HMDs in how they track participant motion: the Pico Neo 2 Eye tracks both position and rotation, and moving the head or the controller results in similar movement in the virtual space, while the Oculus GO tracks only rotation, and the movement of the head and the controller in the virtual space is estimated from rotation.

Before starting the actual data collection, we conducted several pilot measurements both with typically developing children as well as with children exhibiting attention and executive function deficits (see Seesjärvi et al., 2022) to evaluate whether the game play should be conducted in a standing or seated position. Especially younger children who had not previously used VR goggles had problems playing in a standing position (e.g., they tried to reach a wall or table to lean on) and reported mild adverse experiences (feeling dizzy). Although a standing position would have improved sensorimotor contingency, we decided to instruct the children to play in a seated position. This was to assure participant safety and avoid problems potentially influencing data quality (e.g., confounding factors related to stress and adverse effects).

**Fig. 1:**
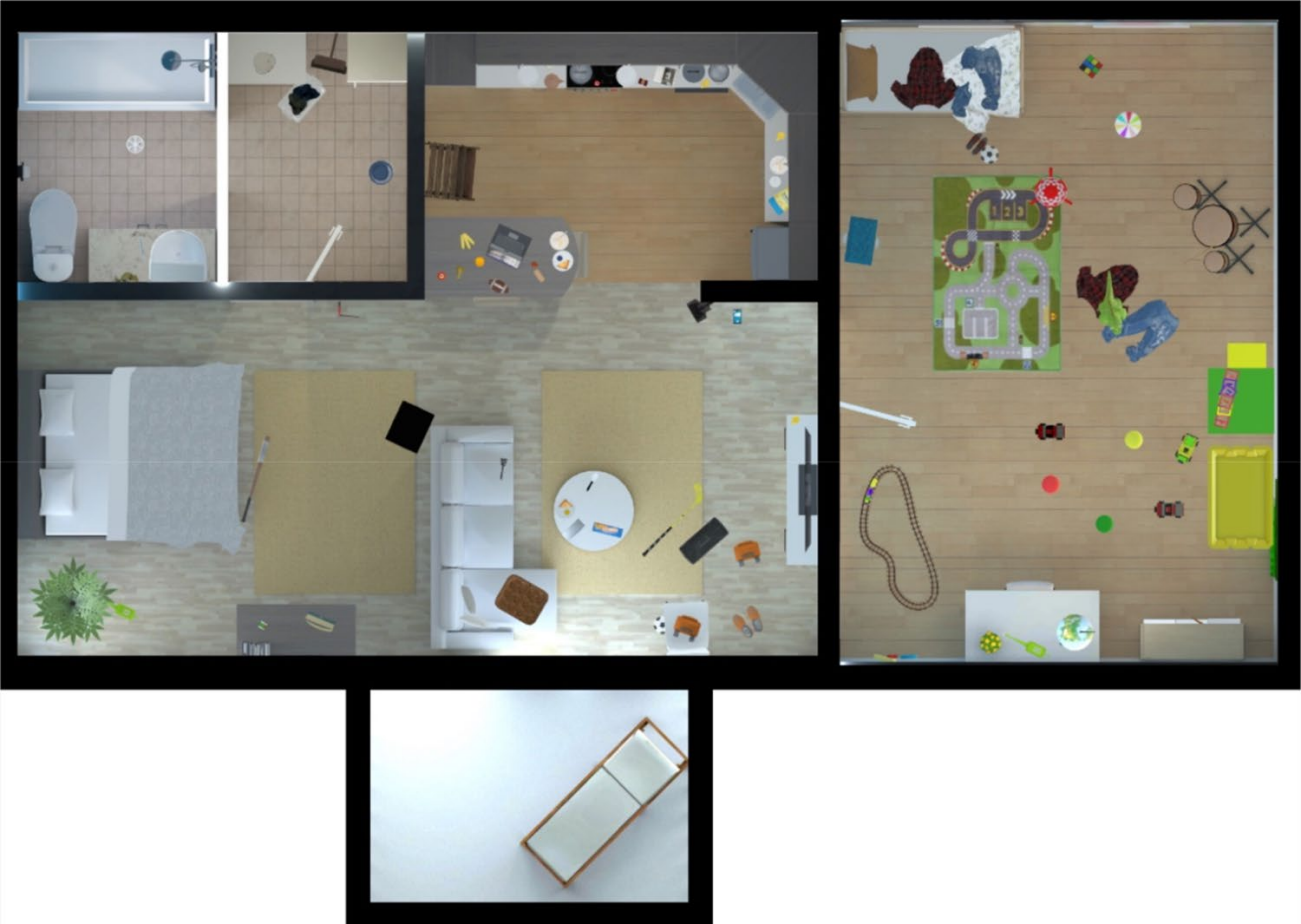
EPELI floor plan (picture copyright Peili Vision, 2022)

The VR environment in EPELI is a rather typical apartment with a living room, an open adult bedroom, an open kitchen, a children’s room, a utility room, a bathroom, and a balcony that is not accessible but visible through windows (see Fig. 1 for a bird’s eye view not shown to the participants). Before the actual task, there is a practice session during which the participant gets familiar with the apartment and practice navigating around, interacting with the objects, and monitoring time with a watch that can be seen when the participant looks down to the hand controller and turns its face toward him/herself. The actual task comprises 13 short everyday scenarios (e.g., “going to school”, “meal preparation”, and “coming back from football training”). A cartoon dragon character guides the participant through the practice session and comes back to give oral instructions before each task scenario, which consist of four to six subtasks (e.g., “turn off the tap that your father forgot to close”, “take your backpack to your room”, “call your mother at two o’clock”). The theme of each scenario is also explicitly given to the participant (e.g., “you are just about to leave for the school”). There are 70 subtasks altogether, 52 of which can be completed at any time, 13 that must be completed at a certain time (time-based subtasks), and five after an external sound cue (event-based subtasks, e.g., a doorbell or cell phone tone). The child is asked to perform the subtasks in the given order, except for the subtasks to be completed at a certain time or after a certain sound cue, but the completion order does not influence the scoring. One task scenario lasts until all subtasks are correctly performed or until the time limit of 90 s is reached. The order of the 13 task scenarios was counterbalanced between the participants so that every other participant conducted them in reverse order. To probe possible distractor effects, seven (forward order) or six (reverse order) scenarios included both auditory distractors (dog barking, child coughing, traffic, music from the radio) and audiovisual distractors (fly buzzing around the player, TV program, water tap left running). These scenarios also contained more task-irrelevant objects. The participants with the scenarios in forward order had different scenarios embedded with the distractions and more task-irrelevant objects than the participants with the scenarios in reverse order, so that at the group level, half of the gameplays for every scenario had distractors and the other half did not. Distractors were on during the whole scenario except for the running tap, TV, and radio, which the participant could turn off. The length of EPELI with the practice session and all 13 task scenarios is on average around 30 min (a maximum of 35 min), depending on the time the participant uses in the practice session and to complete the scenarios.

The eight EPELI measures originally presented in Seesjärvi et al. (2022) were used as the dependent variables.

Total score (the number of all correctly performed subtasks) is a measure of participants’ general prowess in executing multistage goals in naturalistic condition. As it comes from the number of correctly remembered and executed target items, it is considered to have a strong memory component. This is supported by its correlation (*r* = 0.49 in Seesjärvi et al., 2022) with the Repetition task, where participants merely verbally repeat instructions similar to those given in EPELI. However, unlike the Repetition task, achieving high Total score also requires ability to plan and execute the memory task.

Task efficacy taps how well the participants can selectively focus on executing the relevant goals. It is the percentage of relevant actions, i.e., actions that were necessary to successfully complete the subtasks, out of all actions excluding clicks on the waypoints that enable moving around in the environment. Since EPELI environment contains a wealth of irrelevant stimuli that might be distractive or attractive, efficient performance requires ability to inhibit irrelevant stimuli (e.g., a toy that would be nice to play with) that may capture one’s attention or cause interference (e.g., seeing another fruit when the task would be to eat an apple). The definition of task efficacy comes close to a typical definition of selective attention (the ability to selectively process the relevant stimuli and ignore the irrelevant stimuli), but unlike in the typical experimental cognitive tasks, the irrelevant stimuli are designed so that they would be as rewarding and appealing as home environments.

Navigation efficacy measures economy of “walking movement” in the task environment, and it is calculated by dividing the Total score by distance covered, which includes the distance walked and the distance to each manipulated object when used. Efficient navigation requires planning and strategy (e.g., focusing on the relevant tasks and avoiding any additional movements or operations). Navigation efficacy is also correlated with the Repetition task (*r* = 0.51 correlation in Seesjärvi et al., 2022), but even more strongly associated with the ability to avoid irrelevant actions.

Controller motion measures the hand movement during the task. It is considered a general measure of motor activity and calculated as the amount of angular controller movement in degrees. While the level of controller motion does not directly influence task performance, extreme values may reflect neuropsychiatric symptoms (e.g., see associations to hyperactivity in Seesjärvi et al., 2022).

Total actions is a measure of total amount of interaction with the objects in the VR environment. It comprises the number of clicks during the task execution, the number of times hitting the drums in the child’s room by swinging the controller, and the clicks done during the instruction phase of each task scenario. Like task efficacy it captures the irrelevant actions but without taking into account the overall task performance. Hence, it reflects tendency to actively interact with different types of stimuli in the environment. As many stimuli in the EPELI environment are made as attractive and tempt the children for trying them, this measure is expected to reflect impulsive behaviors (see Seesjärvi et al., 2022).

Time-based prospective memory score (TBPM) is the number of time-based subtasks performed within ± 10 s of the given time. It measures prospective memory ability, more precisely accuracy in performing tasks in designated time. Similar measures are widely used in prospective memory research, and they are supposed to reflect ‘when’ aspect in remembering to recall everyday life tasks (e.g., when to leave at school, when to take food out of the oven).

Clock checks is the number of times when the clock has been viewed. It reflects active time-monitoring behavior. Like in real-life, looking at the watch very often interrupts the task performance but occasional monitoring at well-planned intervals (e.g., do I have time to go to the bathroom before the oven is ready) can be important for accurate task performance (especially TBPM performance).

Event-based prospective memory score (EBPM) is the number of event-based subtasks performed within 10 s from the start of the cue. It measures prospective memory ability in the form of responsiveness to external memory cues. External cuing is suggested to be one of the core processes in prospective memory, meaning that in real life, seeing objects or hearing related sounds often triggers us to recall what we were supposed to do. This differs from TBPM also that no strategic monitoring is needed.

### Other tasks

Reasoning abilities were assessed with the Similarities and Matrix reasoning subtests of *the Finnish version of the Wechsler Intelligence Scale for Children* (*WISC-IV*; Wechsler, 2003). Capacity for encoding verbal instructions was assessed with an instruction recall task (see the Repetition task in Seesjärvi et al., 2022), in which the participants verbally repeated a list of instructions like those used in EPELI. Raw scores from each of these three tasks were used in the following data analyses.

### Parent and self-ratings

Parents rated their child’s possible everyday problems in executive functioning with *Behavior Rating Inventory for Executive Functions* (*BRIEF*; Gioia et al., 2000). BRIEF includes eight clinical scales, which form two broader indexes, Behavioral Regulation Index (BRI) and Metacognition Index (MI). The indexes can be summed to obtain an overall score, the Global Executive Composite (GEC). From BRIEF, the raw scores of the BRI, MI, and GEC were used.

After EPELI, the children orally answered to several questionnaires, which were read aloud and filled out by the experimenter. These included *the Simulator Sickness Questionnaire* (Kennedy et al., 1993; see Supplementary Appendix F), a gaming experience questionnaire (see Seesjärvi et al., 2022), a shortened version of *the Presence Questionnaire 3.0* (Witmer et al., 2005; See Supplementary Appendix G), and an object familiarity questionnaire (see Seesjärvi et al., 2022). To assess child’s familiarity with the tasks performed during EPELI, they were asked the question “From a scale of 1 (not at all) to 7 (very much), how much have you performed similar tasks in real life?”. In the following analyses, gaming background (regular gamer or not) and the self-assessed familiarity with the tasks on a scale of 1–7 were used as independent variables.

### Procedure

Measurements were conducted in dedicated rooms at primary schools or at Aalto University, Espoo. For sample A, most tasks (EPELI and questionnaires related to gameplay, the instruction recall task, WISC-IV Matrix reasoning) were completed in a single session except for the WISC-IV Similarities, which was administered at the beginning of a separate session that also included other tasks used in an earlier study (Seesjärvi et al., 2022). For sample B, all tasks were completed in a single session. EPELI was played sitting in a chair that rotated 360° to enable turning in the game effortlessly. Head set position was adjusted if necessary. Sound loudness was the same for all participants and allowed the instructions to be heard clearly.

### Statistical analyses

Statistical analyses and data visualization were done in R version 4.0.3 (R Core Team, 2020) with the additional packages data.table (Dowle & Srinivasan, 2021), stringr (Wickham, 2019), stringi (Gagolewski, 2020), dplyr (Wickham et al., 2021), ggplot2 (Wickham, 2016), gridExtra (Auguie, 2017), patchwork (Pedersen, 2020), and psych (Revelle, 2020).

First, the two samples (A and B) were compared regarding age, gender, average parental income, and average parental education. As there were no differences between the samples on these background variables (see Supplementary Appendix A), the samples were combined for the statistical analyses. The data were complete except for one participant, who found the instruction recall task oppressive and did not finish it.

In the outlier analyses, the EPELI measures were checked for univariate outliers (± 3 SDs from the group mean). As a result, three participants were removed from the final sample, as all analyses included EPELI (final *N* = 77). Also the BRIEF measures (GEC, BRI, MI) were checked for univariate outliers, and based on the results one more participant was excluded from the analyses that included BRIEF. After that, the data were checked for participants with multivariate outliers (Mahalanobis distance, *χ*^2^ using *α* level *p* < 0.001) in the same measures using function mahalanobis from psych package, but none were found. The internal consistency of each EPELI measure across scenarios was assessed with the reliability measure of Cronbach’s alpha using functions alpha and alpha.ci from psych package, and reliabilities above 0.7 were considered acceptable (Nunnally & Bernstein, 1994).

The effects of background factors (age, gender, gaming background, familiarity with the tasks, and the HMD used) on the EPELI measures were examined with general linear models using function lm from base R package stats. There was no collinearity between the independent variables apart from more boys playing regularly than girls (45 boys playing regularly and 1 not, 22 girls playing regularly and 9 not, Fisher’s exact *p* < 0.001). The best fitting models were determined by three methods (forward, backward, and combination) using function step in base R package stats. In the forward method, the fitting started from a null model with no independent variables and proceeded by stepwise addition of the independent variable, the addition of which resulted in the largest decrement in the Akaike Information Criterion (AIC), to the next model until the addition of the next independent variable would have increased the AIC. In the backward method, the fitting started from a full model with all independent variables and proceeded by removing one independent variable at a time using the same rule than in the forward method and stopping when the removal of the next independent variable would have resulted in higher AIC. The combination method proceeded like the forward method, but at each step, one independent variable was removed while another was added, and ended when the next model would have had a higher AIC. All three selection methods resulted in the same models except for the analyses with Clock checks as the dependent variable, where the model that resulted from starting from the null model was chosen, since it had lower AIC than the model that resulted from starting from the full model. The full models are presented in Supplementary Appendix C. On all analyses, the same independent variables yielded statistically significant effects in the best fitting models and full models. To analyze the effect of child’s capacity of encoding verbal instructions on EPELI performance, general linear models with each EPELI measure at a time as the dependent variable and the instruction recall task raw score as the independent variable were estimated. The relationships between EPELI and parent-reported difficulties in executive function were examined by calculating correlations between the EPELI measures and BRIEF measures (GEC, BRI, MI). To inspect these relationships in greater detail, general linear models with each BRIEF measure at a time as the dependent variable and the EPELI measures as possible independent variables were fitted to the data, and the best fitting models were determined using the three methods described above. For these analyses, all three methods yielded in the selection of the same models.

## Results

### Background characteristics

The background characteristics of the final sample (age range 9.0–13.0 years) are shown in Table 1. The average parental income and education level appeared to be slightly higher than in the general Finnish population of 30–44-year-olds.^1^ The verbal reasoning abilities are on average slightly higher [*t*(77) = 4.41, *p* < 0.001] and the perceptual reasoning abilities are on par [*t*(77) = 0.61, *p* = 0.550] with the norms reported in the Finnish WISC-IV test manual (Wechsler, 2003). The children evaluated that they had done similar tasks fairly often in their everyday life as they did in EPELI and reported very few VR-related sickness symptoms after EPELI.

### Reliability of the EPELI measures

The internal consistency of the EPELI measures is reported in Table 2. The consistency is acceptable (Cronbach’s *α* >= 0.70) for all measures except TBPM and EBPM. When the number of scenarios is reduced, the consistency stays acceptable down to five scenarios for total actions, to seven scenarios for task efficacy and controller motion, and to 11 scenarios for navigation efficacy and clock checks but drops under 0.7 for total score for eleven scenarios (see Supplementary Appendix B). With regard to possible improvements to the internal consistency through task manipulations, we observed that dropping out the scenario that is least consistent with the others (the scenario number 2) increases the Cronbach’s *α* of total score to 0.73. The average consistency of all eight measures is 0.71. If TBPM and EBPM measures, which form a part of Total score are not examined separately, the average consistency is 0.79.

**Table 2:** Internal consistency (Cronbach’s alpha) of the EPELI measures

Measure	*α* (95% CI)^a^
Total score	0.70 [0.59, 0.79]
Task efficacy	0.83 [0.77, 0.88]
Navigation efficacy	0.74 [0.65, 0.81]
Controller motion	0.88 [0.85, 0.92]
Total actions	0.87 [0.82, 0.91]
TBPM	0.59 [0.45, 0.71]
Clock checks	0.72 [0.62, 0.80]
EBPM	0.33 [0.13, 0.54]

*N* = 77

^a^A Bootstrap 95% Confidence Interval. TBPM, time-based prospective memory score. EBPM, event-based prospective memory score

### The associations between background factors and EPELI performances

**Fig. 2:**
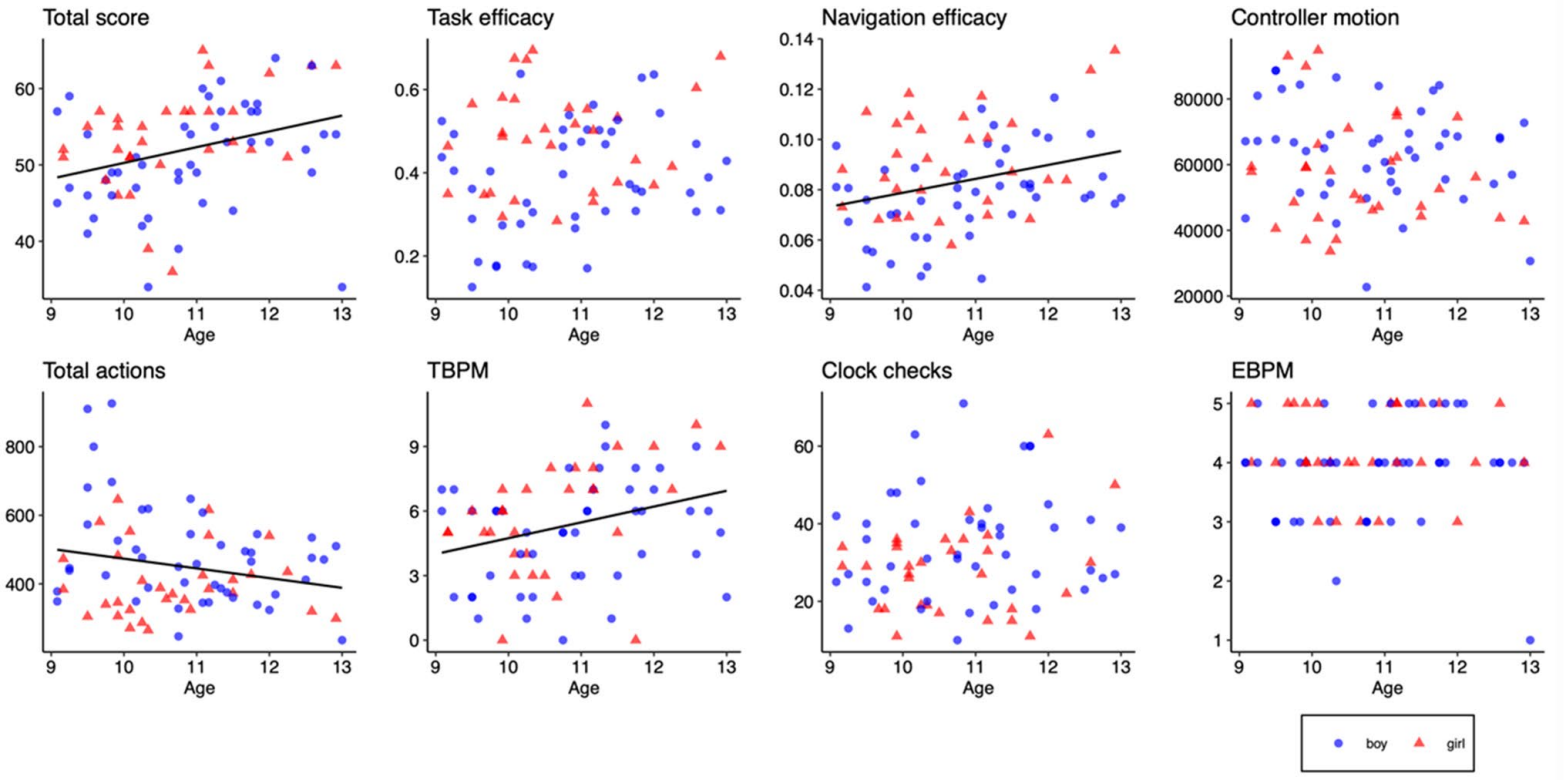
Scatter plots of the EPELI measures per age. The regression line between an EPELI measure and age is shown on those plots where that association was statistically significant

The best fitting linear models with each EPELI measure as the dependent variable and the background factors as the independent variables are presented in Table 3 (see Supplementary Appendix C for the full models). There were statistically significant gender differences in five measures with girls obtaining higher total scores, having higher task and navigation efficacies, and performing fewer total actions and clock checks. Regarding age, older children had significantly higher total and TBPM scores, navigated more efficiently and performed fewer actions. As there were both gender and age effects on total score, navigation efficacy, and total actions, additional models that included interaction between these variables were fitted, but no significant interactions were found. Scatter plots with EPELI measures per age are shown in Fig. 2 (for descriptive statistics, see Supplementary Appendix I). It is worthwhile to note, that in all models the amount of variance explained (Adj. *R*^2^) is fairly low (4–20%), which indicates relatively weak associations between the dependent variable and independent variables.

**Table 3:** The best fitting linear models with each EPELI measure as the dependent variable and age, gender, gaming background, familiarity of the tasks, and the type of HMD as independent variables

Dependent variable	Independent variable	Estimate (*β*)	SD	*t*	*p*	△AIC	*R*^2^	Adj. *R*^2^
Total score	(Intercept)	21.253	8.166	2.60	0.011*	− 3.77	0.192	0.159
	Gender	4.524	1.615	2.80	0.007**			
	Age (years)	2.326	0.700	3.33	0.001**			
	Gaming background	4.272	2.344	1.82	0.072			
Task efficacy	(Intercept)	0.138	0.153	0.90	0.371	− 3.04	0.185	0.151
	Gender	0.099	0.029	3.34	0.001**			
	Age (years)	0.024	0.013	1.77	0.082			
	HMD	− 0.051	0.030	− 1.74	0.085			
Navigation efficacy	(Intercept)	0.010	0.021	0.46	0.645	− 2.97	0.220	0.199
	Gender	0.014	0.004	3.35	0.001***			
	Age (years)	0.006	0.002	3.25	0.002**			
Controller motion	(Intercept)	92,835	18,483	5.02	< 0.001***	− 5.33	0.073	0.048
	Gender	− 7095	3580	− 1.98	0.051			
	Age (years)	− 2664	1685	− 1.58	0.118			
Total actions	(Intercept)	836.820	157.34	5.32	< 0.001***	− 5.01	0.137	0.114
	Gender	− 85.100	30.480	− 2.79	0.007**			
	Age (years)	− 32.570	14.340	− 2.27	0.026*			
TBPM	(Intercept)	− 3.586	2.921	− 1.23	0.223	− 2.16	0.130	0.107
	Age (years)	0.787	0.266	2.95	0.004**			
	Gender	1.038	0.566	1.83	0.070			
Clock checks	(Intercept)	34.696	1.919	18.08	< 0.001***	− 3.02	0.055	0.042
	Gender	− 6.309	3.025	− 2.09	0.040*			
EBPM	(Intercept)	3.870	0.118	32.79	< 0.001***	− 6.42	0.039	0.026
	Gender	0.324	0.186	1.74	0.086			

*N* = 77

*** *p* < 0.001, ** *p* < 0.01, * *p* < 0.05

For gender, girl = 1 and boy = 0. Age in years. For gaming background, regular gaming = 1, no regular gaming = 0. For HMD, Pico Neo 2 Eye = 1, Oculus GO = 0. △AIC, change in the Akaike Information Criterion from the full model

To consider the associations presented above in greater detail, the best models were redone with the instruction recall task raw score as an additional independent variable (Supplementary Appendix D). In these models, instruction recall task performance influences total score, task efficacy, navigation efficacy, and EBPM. Compared to the models presented above, the gender effect on total score and the age effect on total actions become non-significant, whereas in controller motion, the gender effect becomes significant, with boys moving more than girls.

Gaming background, familiarity of the task contents, and HMD type had no statistically significant effect on any of the EPELI measures. Even though the HMD type did not affect EPELI performance, there was a difference in the perceived hand controller quality as the Pico Neo 2 Eye users reported fewer problems than the Oculus GO users (see Supplementary Appendix G).

### The effect instruction recall performance on the EPELI measures

Table 4 shows the effect of the instruction recall task on each EPELI measure. The performance in the instruction recall task explains interindividual differences in total score, task efficacy, navigation efficacy, Total actions, and EBPM (*R*^2^ = 0.07–0.21).

**Table 4:** The linear models with each EPELI measure as the dependent variable and the instruction recall task as an independent variable

Dependent variable	Independent variable	Estimate (*β*)	SD	*t*	*p*	*R*^2^	Adj. *R*^2^
Total score	(Intercept)	42.645	2.352	18.13	< 0.001***	0.194	0.183
	Instruction recall task	0.309	0.073	4.23	< 0.001***		
Task efficacy	(Intercept)	0.245	0.049	4.95	< 0.001***	0.162	0.151
	Instruction recall task	0.006	0.002	3.79	< 0.001***		
Navigation efficacy	(Intercept)	0.058	0.007	8.23	< 0.001***	0.160	0.149
	Instruction recall task	0.001	< 0.001	3.76	< 0.001***		
Controller motion	(Intercept)	65,541	6151	10.65	< 0.001***	0.006	− 0.007
	Instruction recall task	− 133	191	− 0.69	0.491		
Total actions	(Intercept)	593.161	52.119	11.40	< 0.001***	0.099	0.087
	Instruction recall task	− 4.633	1.621	− 2.86	0.006**		
TBPM	(Intercept)	3.926	0.990	3.965	< 0.001***	0.030	0.017
	Instruction recall task	0.047	0.031	1.523	0.132		
Clock checks	(Intercept)	29.226	5.238	5.580	< 0.001***	0.005	− 0.008
	Instruction recall task	0.100	0.163	0.617	0.539		
EBPM	(Intercept)	3.340	0.297	11.237	< 0.001***	0.073	0.060
	Instruction recall task	0.022	0.009	2.417	0.018*		

*N* = 76

*** *p* < 0.001, ** *p* < 0.01, * *p* < 0.05

### The associations between the EPELI measures and different domains of parent-reported executive function problems

The correlations between EPELI and BRIEF measures are shown in Table 5. The GEC is negatively associated with both task and navigation efficacy. Regarding the two indexes that comprise the GEC, BRI has a negative correlation with task and navigation efficacy and a positive correlation with total actions, while MI only correlates negatively with task efficacy. The instruction recall task is negatively associated with BRI, but not with other BRIEF measures (see Supplementary Appendix H). Table 6 shows the best fitting linear models with each BRIEF measure as the dependent variable and EPELI measures as the independent variables. For GEC and MI, the best model includes task efficacy as the sole independent variable, and thus the *R*^2^ of these models is identical to the squares of corresponding correlations in Table 5. For BRI, the best fitting model included Task efficacy and EBPM as the independent variables and yielded adjusted *R*^2^ of 0.156.

**Table 5:** Correlations between EPELI and BRIEF measures

	BRIEF		
	GEC	BRI	MI
EPELI			
Total score	− 0.17	− 0.15	− 0.17
Task efficacy	− 0.33**	− 0.34*	− 0.29*
Navigation efficacy	− 0.29*	− 0.31*	− 0.24
Controller motion	0.14	0.21	0.09
Total actions	0.27	0.40*	0.18
TBPM	− 0.11	− 0.06	− 0.12
Clock checks	0.15	0.09	0.16
EBPM	− 0.22	− 0.20	− 0.21

*N* = 76

*GEC* global executive composite, *BRI* behavioral regulation index, *MI* metacognition index

FDR correction. ** *p* < 0.01, * *p* < 0.05

**Table 6:** The best fitting linear models with each BRIEF measure as the dependent variable and the eight EPELI measure as the possible independent variable

Dependent variable	Independent variable	Estimate (*β*)	SD	*t*	*p*	*R*^2^	Adj. *R*^2^
BRIEF GEC	(Intercept)	117.061	5.645	20.78	< 0.001***	0.111	0.100
	Task efficacy	− 39.115	12.834	− 3.05	0.003**		
BRIEF BRI	(Intercept)	32.486	3.704	8.77	< 0.001***	0.178	0.156
	Task efficacy	0.015	0.004	3.53	< 0.001***		
	EBPM	− 1.012	0.724	− 1.40	0.166		
BRIEF MI	(Intercept)	76.027	4.227	17.99	< 0.001***	0.086	0.073
	Task efficacy	− 25.280	9.610	− 2.63	0.010*		

*N* = 76

*** *p* < 0.001, ** *p* < 0.01, * *p* < 0.05

## Discussion

There is a call for tasks modeled after real-life functions, as these could provide better predictive validity and clinical applicability than tasks that target single cognitive domains (Burgess et al., 2006; Parsons et al., 2017). The aim of this study was to evaluate some key properties of EPELI, a new open-ended virtual reality task for the assessment of goal-oriented behavior (Seesjärvi et al., 2022), which is required to warrant its future use in research and clinical work. The internal consistency was found to be acceptable for six out of eight EPELI measures (Cronbach’s *α* = 0.70–0.88). There were several age and gender differences in EPELI measures that favored older children and girls, whereas no statistically significant effects attributable to previous user experience, task familiarity, or VR technology were found. The ability to encode verbal instructions was related to interindividual differences on several EPELI measures. As for the relationship between EPELI measures and parent-rated problems in executive function, some similar associations were found as in Seesjärvi et al. (2022), although the correlations were weaker in the present study, which did not include a clinical group. Below, we discuss each finding in greater detail.

### Reliability of EPELI

Although internal consistency is an essential aspect of any new test measure, estimates of internal consistency are yet to be reported for many of the new VR-based neuropsychological tasks (e.g., Barnett et al., 2021; Chicchi Giglioli et al., 2021; Ouellet et al., 2018). In the present study, six out of eight EPELI measures showed acceptable internal consistency (Cronbach’s *α* = 0.70–0.88) in the analysis with all 13 scenarios. The average consistency of these eight measures was 0.71. If TBPM and EBPM measures, which are included in Total score, are excluded, the average consistency was 0.79. A roughly similar level of reliability (*α* = 0.79) has been reported for the VR-EAL task (Kourtesis et al., 2021). The internal consistency was highest for the measures based on the most datapoints (controller motion and total actions) and lowest for the measures with the fewest datapoints (time-based and event-based prospective memory). For prospective memory measures, a long inter-item interval is often desired, which limits the number of datapoints and thus the reliability of the measures (McDaniel & Einstein, 2007). Besides internal consistency, test–retest reliability of a prospective memory measures is also important to establish (see e.g., Mioni et al., 2015; Zuber et al., 2021). Thus, this should be probed in future studies with EPELI.

To explore the internal consistency of shorter, more clinically viable EPELI versions, we re-examined the alpha coefficients while dropping out one task scenario at a time. Some of the EPELI measures showed acceptable internal consistency even when about half of the task scenarios were cut out. Thus, the use of a shortened version of EPELI would mean that one should also cut down the relevant outcome variables. According to the present data, a useful compromise could be reached with seven task scenarios, for which three outcome measures (task efficacy, controller motion, and total actions) still show acceptable internal consistency. Alternatively, one could reduce the total duration of the task by shortening the maximum duration of each scenario. However, it remains to be seen how this would affect the reliability coefficients.

It should be noted that in this first iteration of EPELI we chose to use a maximum number of scenarios possible when aiming for a total task duration of approximately 30 min. The internal consistency of EPELI could further be improved by dropping out those scenarios that are less consistent with the others. For example, dropping out the scenario that is least consistent with the others would increase the Cronbach’s *α* of total score to 0.73. This information can be used when designing future EPELI versions.

### The effects of age, gender, and other background variables on EPELI performance

*Age effects*: Executive functions continue to develop until at least early adulthood, and the age range of our participants (9–13 years), albeit rather narrow, is an important period in this maturation process (Casey et al., 2005). One could thus expect to find age effects but due to the novelty of EPELI and the variety of its measures, which range from attentional control to motor activity, it was important to examine the age differences for each measure.

As hypothesized, it was found that older children attained higher Total scores and Navigation efficacy. Given the age effects observed on other types of executive function tasks (Best & Miller, 2010; Klenberg, 2015; Vuontela et al., 2013) and prospective memory (Ballhausen et al., 2019; Mahy et al., 2014), these findings were quite expected. A similar result was also assumed for task efficacy, but this was not the case. One could speculate whether observing age effects in total scores but not in task efficacy could reflect differences in the developmental trajectories of cognitive functions within the present limited age range. For instance, total score relies more on the memory domain and task efficacy on the attention domain.

We also found that older children performed fewer actions overall. As the number of actions required by the tasks is constant and rather low compared to the average number of actions performed by the children,^2^ this decrease indicates less task-irrelevant, exploratory behavior. This could reflect diminished impulsivity with increasing age. This interpretation is in line with our current result that total actions correlate with parent-rated problems of behavioral regulation (BRIEF BRI). It also concurs with our previous finding that ADHD children, prone to impulsive behavior, perform more actions in EPELI than typically developing children (Seesjärvi et al., 2022). Similar age-related differences have been observed in studies of response inhibition (Best & Miller, 2010; Klenberg, 2015). Finally, in our previous EPELI study (Seesjärvi et al., 2022), we did observe a clear correlation (*r* = 0.48) between total actions and ADHD symptoms of impulsivity. Total actions here also correlated significantly (*r* = 0.40) with the Behavioral Regulation Index derived from the parent-rated BRIEF questionnaire. However, another explanation is that the age difference in total actions reflects better memory of the task instructions in older children, lessening the need for exploration to find out forgotten subtasks. Indeed, when instruction recall performance was controlled for in a post hoc analysis, there was no longer an age effect on total actions.

Older children appear to perform better than younger children in the EPELI TBPM but not EBPM subtasks. While TBPM subtasks require accurate monitoring of time while being engaged in other activities, EBPM subtasks rely on external triggers and thus load much less on the executive system. In line with this, Kourtesis and MacPherson (2021b) found at in VR-EAL, EBPM performance is best predicted by delayed recognition performance and TBPM by planning ability. In the current study, the low number of items (5) and ceiling rate performances can explain the lack of age effects on EBPM. The appearance of an age effect on TBPM tasks, in turn, could reflect the development of time perception (Droit-Volet, 2013) or related cognitive functions such as working memory updating and performance monitoring (Voigt et al., 2014). As there was no age effect in overt time monitoring as measured by clock checks, our findings could well be related to the development of underlying cognitive functions.

*Gender effects*: In line with Barnett et al. (2007) who reported gender effects in attention tasks of typically developing children, we found higher scores for girls in several measures reflecting attentional-executive functions (task efficacy, navigation efficacy, total score), and they also performed fewer actions than boys. This pattern of results concurs with the findings that brain maturation follows different trajectories in girls than and boys in this age group (Brenhouse & Andersen, 2011; Giedd et al., 2012; Lenroot et al., 2007). According to the present results, girls were approximately 2.0–2.7 years ahead of boys in their task performance in the measures in which gender differences were observed. There could also be more specific mechanisms underlying gender differences on certain EPELI measures. For example, post hoc analyses indicated that the gender difference in total score was no longer present when instruction recall performance was controlled for. This suggests that the higher accuracy in girls may be due to superior ability in memory, and not in the actual execution of the task. There were no interactions between age and gender, which suggests that the maturation processes discussed above may not differ considerably between boys and girls, at least with respect to the indicators used in the present study.

*The effects of other background variables*: Besides age and gender, we examined the associations between the instruction recall task, gaming background, HMD-type, and familiarity of the task contents on EPELI measures. Based on findings reported in Seesjärvi et al. (2022) and evidence of the role played by episodic memory in prospective memory task outcomes (Kliegel et al., 2008), we expected that instruction recall performance would explain part of the variance in the EPELI tasks, especially regarding those measures that have a strong memory component. This was indeed the case, as the ability to verbally recall instructions was associated with five out of eight EPELI measures. However, it explained only 6–20% of the variability on these EPELI measures, highlighting the role of task execution besides the memory component in EPELI performance.

Gaming background has been reported as a factor that might influence task performance in various types of computerized cognitive tasks (Bediou et al., 2018), but no effects were found in the present study. The finding is in line with that of Kourtesis et al. (2021), who found no performance differences between gamers and non-gamers in VR-EAL, a paradigm also implemented with immersive VR. It has been suggested that in immersive VR, where the interaction with the environment is realistic and intuitive, the effects of gaming background might be less prominent than in non-immersive computerized tasks (Kourtesis et al., 2021). The task contents in EPELI were designed so that they would be familiar to all participants. High familiarity ratings were indeed reported by our participants, and familiarity had no effect on EPELI measures. Finally, as expected, the two different HMDs provided highly similar results, even though there was a difference in the perceived hand controller quality. Thus, small differences between the displays and movement sensors should not influence the EPELI measures. This is a positive finding considering that manufacturers may provide up to one or two updates in HMDs per year.

### The associations between EPELI measures and parent-reported executive function problems

This study replicated the previously reported (Seesjärvi et al., 2022) associations between EPELI efficacy measures and parent-reported executive function problems as evaluated with the BRIEF questionnaire total score (GEC). The present replication is important in showing that this association also exists in typically developing children. EPELI efficacy measures also showed highest correlations with BRIEF GEC in the study by Seesjärvi et al. (2022). Other associations found in the previous study, with a sample including both ADHD children and typically developing children, did not reach statistical significance in this sample, nor did the measures of specific prospective memory domains (TBPM, EBPM, and monitoring). It appears a reasonable inference that the global measures reflecting how the task was performed rather than the level of performance in a specific domain would be the most sensitive indicators of everyday problems. However, further evidence is clearly needed to confirm this preliminary finding.

In this study, we further examined the associations between EPELI measures and two BRIEF indexes, namely the Behavioral Regulation Index (BRI) and Metacognition Index (MI). There were some associations between EPELI and BRI (total actions and navigation efficacy) that were not observed when EPELI measures were correlated with MI. It does make sense that both total actions as well as navigation efficacy could reflect self-regulation abilities (e.g., inhibition of irrelevant actions) rather than metacognitive functions. This could be further tested by conducting item-level analyses clarifying the behavioral processes associated with these two EPELI measures.

Finally, examining the associations between each BRIEF measure and several EPELI measures revealed that the BRIEF scores were best predicted by task efficacy alone or, in the case of BRI, task efficacy and EBPM. Taken as whole, the results regarding the associations of EPELI and BRIEF measures suggest that in EPELI, behavioral problems are predicted by lower task efficacy, and, in the case of regulatory problems, also by more difficulties in memorizing cue-triggered prospective tasks or failure to execute them. They also provide support for the ecological validity (veridicality) of EPELI.

The correlations between performance-based and rating measures of executive function are generally low, and it has therefore been suggested that these measures assess different mental constructs (Toplak et al., 2013). The correlation estimates acquired here cannot be tested straightforwardly against those reported in earlier studies. However, in an earlier study (Seesjärvi et al., 2022), the correlation between EPELI task efficacy and BRIEF was stronger than the correlations between BRIEF and other performance-based measures, except for CPT reaction time variability. This suggests that in contrast to the previously used highly structured tasks with highly restricted stimuli and responses, open-ended tasks that simulate real-life contexts and functions could provide objective measures more closely associated with the constructs assessed by the rating measures.

### Limitations of the study and future directions

There are some potential limitations to be considered when interpreting the present findings. First, even though our sample size is larger than in most previous VR studies examining attentional-executive functions (see, e.g., Parsons et al., 2019; Kim et al., 2021), it is somewhat limited considering the sampling distributions. The age distribution in this sample is relatively narrow. Since the participants were recruited so that the families contacted us based on the advertisements, a sampling bias may be present. Although we attempted to collect data from schools at areas with varying socio-economic backgrounds, the average parental education and income remained slightly over the population mean. This and other limitations of the representativeness of the sample should be considered, especially when evaluating the usefulness of this data as a reference population. Second, when using BRIEF as a criterion measure for everyday problems, it should be considered that, unlike EPELI, BRIEF is affected by subjective bias and the evaluation given encompasses a longer time. It is also possible that the underlying constructs that executive function questionnaires and tasks tap are not quite the same. Questionnaires concern typical performance in daily life, while laboratory measures such as EPELI represent an explicit testing situation where the motivation is high due to the engaging task and competitive configuration (see Toplak et al., 2013). Such factors might thus influence the correlational analyses. Third, in this validation study we decided to focus on average performance over multiple scenarios. However, EPELI does provide precise item-level raw data regarding any object interaction or movement from one location to another. There are thus opportunities for more detailed analyses in future studies. Fourth, gaming background, previous familiarity with task contexts, and perceived presence are complex phenomena and only a limited investigation of related influences on EPELI performance was possible in the scope of this study. Future studies should also examine whether a specific kind of gaming background (e.g., expertise on first-person three-dimensional games vs. two-dimensional strategy games) is related to EPELI task performances. These possible more fine-grained associations should be a target in future studies with thorough questionnaires of these background factors. The presence questionnaire employed here was modified from the adults’ version, as to our knowledge, there is no such a questionnaire designed specifically for children. The development of such a questionnaire should be a target of future research.

While in this first study with EPELI, we instructed the participants to play the game in a sitting position, with teleporting for navigating in the environment and raycasting to interact with the objects, there are novel opportunities that could be used to further improve the sensorimotor contingency in future research. With modern extended reality (XR) headsets (e.g., Varjo XR-3), it is possible to move in a real room and to interact with the objects (either virtual or real ones) using one’s own hands. However, such approach will require still considerable technical testing before taking into use in clinical studies in children, and remains an impractical option for hospital use as a dedicated room and costly equipment is needed. At the moment, it should be kept in mind that the current setup is not optimal to assess such disorders where motor deficits or visuomotor integration play a central role (e.g., dyspraxia).

Finally, as EPELI could serve as a complementary tool for neuropsychological evaluations, it is necessary to consider some pre-requisite aspects for this. In a joint position paper of the American Academy of Clinical Neuropsychology and the National Academy of Neuropsychology, Bauer et al. (2012) identify eight key issues relevant to the development and use of computerized neuropsychological assessments. These eight issues concern marketing and performance claims; appropriate end-users; hardware/software/firmware issues; privacy/data security/identity verification/testing environment; reliability and validity; cultural/experiential/disability factors; use of computerized testing and reporting services; the need to control for response validity and effort. As pointed out by Kourtesis and MacPherson in their work on VR-EAL (2021a), immersive VR paradigms can meet these criteria, and therefore become valuable additions for neuropsychological assessments. The results shown here and in a previous study (Seesjärvi et al., 2022) address some of these areas, such as reliability (internal consistency), ecological validity, potential sickness symptoms, and discriminant validity between children with ADHD and typically developing controls. Some aspects however, related for example to the end-user requirements and reporting of the results, cannot be evaluated yet, as an end-user interface for clinical work has not been yet developed. After all necessary parts of software have been developed and suitable hardware alternatives have been identified, future research should evaluate the rest of these areas collectively to ensure the feasibility of EPELI for broader clinical use.

## Conclusions

This study set out to examine several key properties of a novel virtual reality task, EPELI, that has been developed to measure goal-directed behavior in naturalistic conditions. Our findings demonstrate acceptable internal consistency for six out of eight EPELI measures. Moreover, for four measures, internal consistency remained adequate even when the number of scenarios was cut to approximately half. EPELI performance was also associated with interindividual variability in everyday attentional executive problems. This demonstrates the opportunities of ecologically valid VR tasks in objective measurement of children’s cognitive functions in situations resembling those where they manifest in real life. Moreover, we report the effects of age and gender on EPELI performance. Our results suggested that girls are better able to focus selectively on the relevant tasks, plan their route in the game more efficiently, and successfully executed higher number of instructed tasks. As task scenarios in EPELI were selected to represent relevant aspects of children’s daily life, this type of games may provide a more direct way of measuring individual differences in cognitive abilities in contextually relevant and engaging conditions. Both age and gender are also important factors to consider for future studies in different child populations. In conclusion, we provide evidence of the reliability and validity of a new virtual reality tool for ecologically valid objective assessment of attention, executive functions, and prospective memory in children.

## Supplementary Information

Below is the link to the electronic supplementary material.

Supplementary file1 (DOCX 59 KB)

## Data Availability

In compliance with the research permission by the Ethics Committee of the Helsinki University Hospital, supporting data for this study is not available.

## Footnotes

1 The average income before tax was 3406 €/m in 2016 for adults aged 30–44 (Official Statistics of Finland, 2022a). The average education for adults aged 30–44 is 2.3 when converted to the scale used in this study (Official Statistics of Finland, 2022b).

2 To obtain full score of 70, 107 actions plus moving actions are required. In the present sample, the children performed on average 451 actions, of which on average 150 were related to moving.
