# Supplementary material for: EPELI: a novel virtual reality task for the assessment of goal-directed behavior in real-life contexts
Source: Psychol Res. 2022 Nov 23;87(6):1899–916. doi: 10.1007/s00426-022-01770-z (PMC10366028; doi:10.1007/s00426-022-01770-z)
Supplement: Supplementary file 1 — Supplementary file1 (DOCX 59 KB) [file 426_2022_1770_MOESM1_ESM.docx]

**Supplementary Appendix A.**

Background characteristics of the subsamples.

|  | *SAMPLE A (n = 51)* | | *SAMPLE B (n = 29)* | |  |  |
| --- | --- | --- | --- | --- | --- | --- |
| Variable | *M* | *SD* | *M* | *SD* | Test statistic | *p* |
| Age | 10 y 10 m | 1 y 1 m | 10 y 9 m | 1 y 1 m | *t(58) = 0.45* | .656 |
| Gender (boy / girl) | 31 / 20 |  | 18 / 11 |  | Fisher’s Exact test | .999 |
| Parental education** | 2.8 | 0.47 | 2.8 | 0.41 | *t(65) = -0.21* | .834 |
| Parental income: |  |  |  |  |  |  |
| *less than 1500 €/m* | 0 |  | 0 |  | Fisher’s Exact test | .252 |
| *1500–2200 €/m* | 3 |  | 0 |  |  |  |
| *2200–3000 €/m* | 8 |  | 3 |  |  |  |
| *3000–4000 €/m* | 17 |  | 8 |  |  |  |
| *over 4000 €/m* | 23 |  | 19 |  |  |  |
| *Note*. * Before tax per adult. ** Average parental education with 1 = Comprehensive school, 2 = High school / Vocational school, 3 = University degree or equivalent. | | | | | | |

**Supplementary Appendix B.**

*Internal consistency (Cronbach’s alpha) of each EPELI measure for task sets with different number of scenarios.*

|  | Number of scenarios | | | | | | | | | | | |
| --- | --- | --- | --- | --- | --- | --- | --- | --- | --- | --- | --- | --- |
| Measure | 13 | 12 | 11 | 10 | 9 | 8 | 7 | 6 | 5 | 4 | 3 | 2 |
| Total score | .70 | .69 | .67 | .63 | .63 | .60 | .59 | .52 | .49 | .42 | .33 | .33 |
| Task efficacy | .83 | .82 | .80 | .80 | .78 | .77 | .75 | .69 | .64 | .52 | .52 | .32 |
| Navigation efficacy | .74 | .73 | .70 | .69 | .69 | .65 | .63 | .58 | .53 | .45 | .49 | .38 |
| Controller motion | .88 | .88 | .86 | .86 | .82 | .79 | .76 | .68 | .66 | .60 | .61 | .47 |
| Total actions | .87 | .86 | .85 | .86 | .84 | .83 | .81 | .76 | .77 | .69 | .65 | .73 |
| TBPM | .59 | .58 | .55 | .55 | .55 | .54 | .57 | .54 | .53 | .49 | .46 | .38 |
| Clock checks | .72 | .72 | .70 | .69 | .67 | .59 | .53 | .42 | .39 | .56 | .52 | .46 |
| EBPM | .33 | .33 | .33 | .30 | .30 | .33 | .33 | .09 | .09 | - | - | - |
| *Note*. *N* = 77. TBPM, time-based prospective memory score. EBPM, event-based prospective memory score. | | | | | | | | | | | | |

**Supplementary Appendix C.**

*The full linear models with each EPELI measure as the dependent variable and age, gender, gaming background, familiarity of the tasks, and the type of HMD as independent variables.*

| Dependent variable | Independent  variable | Estimate (β) | *SD* | *t* | *p* | *R^2^* | Adj. *R^2^* |
| --- | --- | --- | --- | --- | --- | --- | --- |
| Total score | (Intercept) | 20.018 | 8.875 | 2.26 | .027* | .194 | .137 |
|  | Gender | 4.662 | 1.663 | 2.80 | .007** |  |  |
|  | Age (years) | 2.307 | 0.709 | 3.25 | .002** |  |  |
|  | Gaming background | 4.393 | 2.433 | 1.81 | .075 |  |  |
|  | Task familiarity | 0.287 | 0.715 | 0.40 | .689 |  |  |
|  | HMD | -0.344 | 1.562 | -0.22 | .827 |  |  |
| Task efficacy | (Intercept) | 0.062 | 0.176 | 0.35 | .727 | .195 | .138 |
|  | Gender | 0.109 | 0.033 | 3.30 | .002** |  |  |
|  | Age (years) | 0.024 | 0.014 | 1.72 | .090 |  |  |
|  | Gaming background | 0.020 | 0.048 | 0.41 | .680 |  |  |
|  | Task familiarity | 0.012 | 0.014 | 0.85 | .398 |  |  |
|  | HMD | -0.054 | 0.031 | -1.75 | .084 |  |  |
| Navigation efficacy | (Intercept) | -0.001 | 0.024 | -0.03 | .974 | .250 | .197 |
|  | Gender | 0.016 | 0.005 | 3.48 | <.001*** |  |  |
|  | Age (years) | 0.006 | 0.002 | 3.13 | .003** |  |  |
|  | Gaming background | 0.001 | 0.007 | 0.22 | .830 |  |  |
|  | Task familiarity | 0.003 | 0.002 | 1.32 | .193 |  |  |
|  | HMD | -0.005 | 0.004 | -1.07 | .290 |  |  |
| Controller motion | (Intercept) | 91633 | 21518 | 4.26 | <.001*** | .081 | .016 |
|  | Gender | -6331 | 4032 | -1.57 | .121 |  |  |
|  | Age (years) | -2547 | 1720 | -1.48 | .143 |  |  |
|  | Gaming background | 3865 | 5900 | 0.66 | .515 |  |  |
|  | Task familiarity | -744 | 1732 | -0.43 | .669 |  |  |
|  | HMD | -263 | 3787 | -0.07 | .945 |  |  |
| Total actions | (Intercept) | 875.505 | 182.794 | 4.79 | <.001*** | .148 | .088 |
|  | Gender | -84.984 | 34.251 | -2.48 | .016* |  |  |
|  | Age (years) | -31.674 | 14.610 | -2.17 | .034* |  |  |
|  | Gaming background | 18.930 | 50.117 | 0.38 | .707 |  |  |
|  | Task familiarity | -12.688 | 14.717 | -0.86 | .392 |  |  |
|  | HMD | -7.975 | 32.171 | -0.25 | .805 |  |  |
| TBPM | (Intercept) | 10.264 | 17.899 | 0.57 | .568 | .172 | .114 |
|  | Gender | -4.530 | 3.354 | -1.35 | .181 |  |  |
|  | Age (years) | 1.845 | 1.431 | 1.29 | .201 |  |  |
|  | Gaming background | 5.602 | 4.907 | 1.14 | .257 |  |  |
|  | Task familiarity | -0.489 | 1.441 | -0.34 | .735 |  |  |
|  | HMD | 3.577 | 3.150 | 1.14 | .260 |  |  |
| Clock shows | (Intercept) | 10.264 | 17.899 | 0.57 | .568 | .114 | .052 |
|  | Gender | -4.530 | 3.354 | -1.35 | .181 |  |  |
|  | Age (years) | 1.845 | 1.431 | 1.29 | .201 |  |  |
|  | Gaming background | 5.602 | 4.907 | 1.14 | .257 |  |  |
|  | Task familiarity | -0.489 | 1.441 | -0.34 | .735 |  |  |
|  | HMD | 3.577 | 3.150 | 1.14 | .260 |  |  |
| EBPM | (Intercept) | 2.834 | 1.125 | 2.52 | .014* | .058 | -.008 |
|  | Gender | 0.401 | 0.211 | 1.90 | .062 |  |  |
|  | Age (years) | 0.045 | 0.090 | 0.50 | .616 |  |  |
|  | Gaming background | 0.118 | 0.309 | 0.38 | .702 |  |  |
|  | Task familiarity | 0.089 | 0.091 | 0.99 | .328 |  |  |
|  | HMD | -0.065 | 0.198 | -0.33 | .745 |  |  |
| *Note. N = 77.* *** *p* < .001, ** *p* < .01, * *p* < .05. For gender, girl = 1 and boy = 0. Age in years. For gaming background, regular gaming = 1, no regular gaming = 0. For HMD, Pico Neo 2 Eye = 1, Oculus GO = 0. | | | | | | | |

**Supplementary Appendix D.**

*The best fitting linear models from Table 3 with the instruction recall task as an additional independent variable.*

| Dependent variable | Independent  variable | Estimate (β) | *SD* | *t* | *p* | R^2^ | Adj. R^2^ |
| --- | --- | --- | --- | --- | --- | --- | --- |
| Total score | (Intercept) | 22.136 | 7.458 | 2.968 | .004** | .284 | .244 |
|  | Gender | 2.457 | 1.575 | 1.56 | .123 |  |  |
|  | Age (years) | 1.795 | 0.652 | 2.752 | .008** |  |  |
|  | Gaming background | 2.686 | 2.196 | 1.223 | .225 |  |  |
|  | Instruction recall task | 0.237 | 0.076 | 3.1 | .003** |  |  |
| Task efficacy | (Intercept) | 0.093 | 0.147 | 0.631 | .53 | .275 | .235 |
|  | Gender | 0.072 | 0.029 | 2.459 | .016* |  |  |
|  | Age (years) | 0.016 | 0.013 | 1.202 | .233 |  |  |
|  | HMD | -0.057 | 0.029 | -1.976 | .052 |  |  |
|  | Instruction recall task | 0.005 | 0.002 | 3.097 | .003** |  |  |
| Navigation efficacy | (Intercept) | 0.005 | 0.021 | 0.231 | .818 | .286 | .256 |
|  | Gender | 0.011 | 0.004 | 2.674 | .009** |  |  |
|  | Age (years) | 0.005 | 0.002 | 2.749 | .008** |  |  |
|  | Instruction recall task | 0.001 | <0.001 | 2.723 | .008** |  |  |
| Controller motion | (Intercept) | 94879 | 18615 | 5.097 | <.001*** | .085 | .046 |
|  | Gender | -7756 | 3738 | -2.075 | .042* |  |  |
|  | Age (years) | -2857 | 1714 | -1.667 | .100 |  |  |
|  | Instruction recall task | 20 | 196 | 0.103 | .918 |  |  |
| Total actions | (Intercept) | 882.374 | 156.182 | 5.65 | <.001*** | .186 | .152 |
|  | Gender | -70.64 | 31.366 | -2.252 | .027* |  |  |
|  | Age (years) | -28.064 | 14.381 | -1.951 | .055 |  |  |
|  | Instruction recall task | -3.208 | 1.646 | -1.949 | .055 |  |  |
| TBPM | (Intercept) | -3.588 | 2.957 | -1.213 | .229 | .130 | .094 |
|  | Age (years) | 0.854 | 0.594 | 1.438 | .155 |  |  |
|  | Gender | 0.731 | 0.272 | 2.685 | .009** |  |  |
|  | Instruction recall task | 0.023 | 0.031 | 0.74 | .462 |  |  |
| Clock checks | (Intercept) | 29.179 | 5.071 | 5.755 | <.001*** | .080 | .055 |
|  | Gender | -7.641 | 3.127 | -2.444 | .017** |  |  |
|  | Instruction recall task | 0.203 | 0.163 | 1.246 | .217 |  |  |
| EBPM | (Intercept) | 3.341 | 0.297 | 11.241 | <.001*** | .086 | .061 |
|  | Gender | 0.184 | 0.183 | 1.006 | .318 |  |  |
|  | Instruction recall task | 0.02 | 0.01 | 2.076 | .041* |  |  |
| *Note.* *N* = 77. *** *p* < .001, ** *p* < .01, * *p* < .05. For gender, girl = 1 and boy = 0. Age in years. For gaming background, regular gaming = 1, no regular gaming = 0. For HMD, Pico Neo 2 Eye = 1, Oculus GO = 0. | | | | | | | |

**Supplementary Appendix E.**

*The correlations of the EPELI measures.*

|  | Total score | Task efficacy | Navigation efficacy | Controller motion | Total actions | TBPM | Clock checks | EBPM |
| --- | --- | --- | --- | --- | --- | --- | --- | --- |
| Total score | 1.00*** | 0.41** | 0.65*** | 0.11 | -0.12 | 0.77*** | 0.21 | 0.60*** |
| Task efficacy |  | 1.00*** | 0.80*** | -0.46*** | -0.81*** | 0.29 | -0.04 | 0.28 |
| Navigation efficacy |  |  | 1.00*** | -0.43** | -0.65*** | 0.53*** | 0.05 | 0.40** |
| Controller motion |  |  |  | 1.00*** | 0.68*** | 0.01 | 0.26 | 0.11 |
| Total actions |  |  |  |  | 1.00*** | -0.09 | 0.11 | -0.12 |
| TBPM |  |  |  |  |  | 1.00*** | 0.34 | 0.32 |
| Clock checks |  |  |  |  |  |  | 1.00*** | -0.01 |
| EBPM |  |  |  |  |  |  |  | 1.00*** |
| *Note. N* = 77. TBPM, time-based prospective memory score. EBPM, event-based prospective memory score.*** *p* <.001, ** *p* < .01, * *p* < .05. | | | | | | | | |

**Supplementary Appendix F.**

*The Child Simulator Sickness Questionnaire.* All questions were answered with three-point scale (No = 0; A little = 1; A lot = 2). These are the original questions (Hoeft et al., 2003). A Finnish translation was employed in the present study.

|  |
| --- |
| Question |
| 1. Do you feel sick? |
| 1. Does your head hurt? |
| 1. Do your eyes hurt? |
| 1. Do you have an upset stomach? |
| 1. Are you dizzy with your eyes open? |
| 1. Are you dizzy with your eyes closed? |
| 1. Are you burping at all? |

**Supplementary Appendix G.**

*The Shortened version of the Presence Questionnaire.* This questionnaire is based on Presence Questionnaire 3.0 (see Witmer, Jerome, and Singer, 2005, for revised factor structure). The first nine questions appear on the original questionnaire as such or slightly differently. If the question has been altered to be more suitable for children or the shortened version, the original form is presented in parentheses. A Finnish translation was used in the present study. The following instruction was read by the experimenter: *“I’m going to ask you some questions regarding the game. You can answer by choosing the best alternative from the scale of 1–7 you can see on the screen. The margin on the left means “no” and the margin on the right means “completely/very much”, the rest of the alternatives are between those.”*

|  | HMD TYPE | | | |  |  |
| --- | --- | --- | --- | --- | --- | --- |
|  | Oculus GO | | Pico Neo 2 Eye | |  |  |
| Question | *M* | *SD* | *M* | *SD* | *t(57)* | *p* |
| 1. How natural did your interactions with the  environment seem? | 4.65 | 1.18 | 4.86 | 1.15 | -0.74 | .667 |
| 1. How much did the environment involve you?  (How much did the visual aspects of the environment  involve you? & How much did the auditory aspects of the environment involve you?) | 5.24 | 1.56 | 5.18 | 1.33 | 0.20 | .844 |
| 1. How natural was the mechanism which controlled  movement through the environment? | 3.88 | 1.75 | 4.57 | 1.67 | -1.73 | .216 |
| 1. How much did your experiences in the virtual  environment seem consistent with your real-world  experiences? | 5.10 | 1.39 | 4.79 | 1.47 | 0.92 | .615 |
| 1. How much did the visual display quality interfere or  distract you from performing assigned tasks or  required activities? | 2.35 | 1.35 | 1.79 | 1.03 | 2.05 | .176 |
| 1. How much did the control devices interfere with the  performance of assigned tasks or with other activities? | 1.71 | 1.19 | 1.11 | 0.42 | 3.24 | .024* |
| 1. How well could you concentrate on the assigned tasks  or required activities? (How well could you concentrate  on the assigned tasks or required activities rather than  on the mechanisms used to perform those tasks or  activities?) | 5.47 | 1.17 | 5.57 | 1.17 | -0.37 | .844 |
| 1. How well could you hear sounds? (How well could you  identify sounds? & How well could you localize sounds?) | 6.73 | 0.64 | 6.82 | 0.48 | -0.68 | .667 |
| 1. Were there moments during the virtual environment  experience when you felt completely focused on the  task or environment? | 4.67 | 1.96 | 3.68 | 1.96 | 2.14 | .176 |
| Three additional questions that were not in the original Presence Questionnaire 3.0: | | | | | | |
| 1. How enthusiastic did you feel about the tasks? | 5.55 | 1.43 | 5.14 | 1.51 | 1.16 | .500 |
| 1. How interesting did the tasks seem to you? | 5.35 | 1.55 | 4.75 | 1.40 | 1.73 | .216 |
| 1. How much effort did you put into your performance? | 6.16 | 0.99 | 6.11 | 1.07 | 0.23 | .844 |
| *Note.* *N* = 77. FDR correction. * *p* < .05. | | | | | | |

**Supplementary Appendix H.**

*Correlations between the instruction recall task and BRIEF measures.*

|  |  | BRIEF | | |
| --- | --- | --- | --- | --- |
|  |  | GEC | BRI | MI |
| Instruction recall task | | -.17 | -.31* | -.09 |
| *Note.* *N* = 75. GEC = Global Executive Composite. BRI = Behavioral Regulation Index.  MI = Metacognition Index. FDR correction. * *p* < .05. | | | | |

**Supplementary Appendix I.**

*Descriptive statistics of EPELI measures for* ***all participants (*N*=77)****.*

|  |  |  | percentile | | | | | | | | |
| --- | --- | --- | --- | --- | --- | --- | --- | --- | --- | --- | --- |
| Measure | *M* | *SD* | 0^th^ | 2.2^th^ | 9.1^th^ | 25^th^ | 50^th^ | 75.1^th^ | 90.9^th^ | 97.7^th^ | 100^th^ |
| Total score (0–70) | 51.91 | 6.92 | 34 | 35.34 | 42.92 | 48 | 52 | 57 | 61.08 | 63.25 | 65 |
| Task efficacy | 0.42 | 0.14 | 0.13 | 0.17 | 0.26 | 0.33 | 0.43 | 0.52 | 0.61 | 0.68 | 0.69 |
| Navigation efficacy | 0.08 | 0.02 | 0.04 | 0.05 | 0.06 | 0.07 | 0.08 | 0.1 | 0.11 | 0.12 | 0.14 |
| Controller motion | 61214 | 15701 | 22727 | 32650 | 41963 | 49769 | 60772 | 69508 | 84169 | 90713 | 94794 |
| Total actions | 450.87 | 138.52 | 236 | 259.1 | 305.92 | 349 | 425 | 526.68 | 621.27 | 827.72 | 926 |
| TBPM (0–13) | 5.32 | 2.56 | 0 | 0 | 2 | 3 | 6 | 7 | 9 | 10 | 11 |
| Clock checks | 32.16 | 13.3 | 10 | 11 | 17 | 23 | 30 | 39.08 | 50.08 | 63 | 71 |
| EBPM (0–5) | 4 | 0.81 | 1 | 2.67 | 3 | 4 | 4 | 5 | 5 | 5 | 5 |
| *Note*. TBPM, time-based prospective memory score. EBPM, event-based prospective memory score. | | | | | | | | | | | |

*Descriptive statistics of EPELI measures for* ***younger (aged 9–10) participants (*n*=34)****.*

|  |  |  | percentile | | | | | | | | |
| --- | --- | --- | --- | --- | --- | --- | --- | --- | --- | --- | --- |
| Measure | *M* | *SD* | 0^th^ | 2.2^th^ | 9.1^th^ | 25^th^ | 50^th^ | 75.1^th^ | 90.9^th^ | 97.7^th^ | 100^th^ |
| Total score (0–70) | 49.06 | 5.6 | 34 | 37.63 | 42 | 46 | 49.5 | 52.78 | 56 | 57.48 | 59 |
| Task efficacy | 0.4 | 0.16 | 0.13 | 0.16 | 0.18 | 0.29 | 0.38 | 0.5 | 0.64 | 0.68 | 0.69 |
| Navigation efficacy | 0.08 | 0.02 | 0.04 | 0.04 | 0.05 | 0.07 | 0.08 | 0.09 | 0.11 | 0.11 | 0.12 |
| Controller motion | 63843 | 17763 | 33625 | 36094 | 40539 | 50921 | 64564 | 78822 | 88681 | 93448 | 94794 |
| Total actions | 484.15 | 173.82 | 265 | 269.36 | 305 | 349 | 442.5 | 579.26 | 696.95 | 913.86 | 926 |
| TBPM (0–13) | 4.29 | 1.98 | 0 | 0.73 | 2 | 3 | 5 | 6 | 7 | 7 | 7 |
| Clock checks | 29.71 | 11.65 | 11 | 12.45 | 18 | 20 | 29 | 35.78 | 47.98 | 53.89 | 63 |
| EBPM (0–5) | 3.91 | 0.75 | 2 | 2.73 | 3 | 3.25 | 4 | 4 | 5 | 5 | 5 |
| *Note*. TBPM, time-based prospective memory score. EBPM, event-based prospective memory score. | | | | | | | | | | | |

*Descriptive statistics of EPELI measures for* ***older (aged 11–13) participants (*n*=43)****.*

|  |  |  | percentile | | | | | | | | |
| --- | --- | --- | --- | --- | --- | --- | --- | --- | --- | --- | --- |
| Measure | *M* | *SD* | 0^th^ | 2.2^th^ | 9.1^th^ | 25^th^ | 50^th^ | 75.1^th^ | 90.9^th^ | 97.7^th^ | 100^th^ |
| Total score (0–70) | 54.16 | 7.08 | 34 | 35.85 | 44.82 | 51.5 | 55 | 58 | 63 | 64.03 | 65 |
| Task efficacy | 0.44 | 0.11 | 0.17 | 0.26 | 0.3 | 0.35 | 0.46 | 0.52 | 0.57 | 0.64 | 0.68 |
| Navigation efficacy | 0.09 | 0.02 | 0.04 | 0.06 | 0.07 | 0.08 | 0.08 | 0.1 | 0.11 | 0.13 | 0.14 |
| Controller motion | 59135 | 13717 | 22727 | 30049 | 43544 | 49628 | 58790 | 68473 | 76010 | 83957 | 84153 |
| Total actions | 424.56 | 96.82 | 236 | 246.16 | 323.29 | 354.5 | 412 | 493.17 | 545 | 617.09 | 648 |
| TBPM (0–13) | 6.14 | 2.7 | 0 | 0 | 2 | 5 | 7 | 8 | 9 | 10.03 | 11 |
| Clock checks | 34.09 | 14.31 | 10 | 10.92 | 16.64 | 24.5 | 33 | 40.54 | 60 | 63.27 | 71 |
| EBPM (0–5) | 4.07 | 0.86 | 1 | 2.85 | 3 | 4 | 4 | 5 | 5 | 5 | 5 |
| *Note*. TBPM, time-based prospective memory score. EBPM, event-based prospective memory score. | | | | | | | | | | | |
